# Plasmonic optical fiber for bacteria manipulation—characterization and visualization of accumulation behavior under plasmo-thermal trapping

**DOI:** 10.1364/BOE.425405

**Published:** 2021-06-08

**Authors:** Jang Ah Kim, Eric M. Yeatman, Alex J. Thompson

**Affiliations:** 1 The Hamlyn Centre, Institute of Global Health Innovation (IGHI), Imperial College London, Exhibition Road, South Kensington, London SW7 2AZ, UK; 2 Department of Electrical and Electronic Engineering, Imperial College London, Exhibition Road, South Kensington, London SW7 2AZ, UK; 3 Surgical Innovation Centre (Paterson Building), Department of Surgery & Cancer, St Mary’s Hospital, Imperial College London, South Wharf Road, London W2 1NY, UK; 4 j.a.kim@imperial.ac.uk; 5 alex.thompson08@imperial.ac.uk

## Abstract

In this article, we demonstrate a plasmo-thermal bacterial accumulation effect using a miniature plasmonic optical fiber. The combined action of far-field convection and a near-field trapping force (referred to as thermophoresis)—induced by highly localized plasmonic heating—enabled the large-area accumulation of *Escherichia coli*. The estimated thermophoretic trapping force agreed with previous reports, and we applied speckle imaging analysis to map the in-plane bacterial velocities over large areas. This is the first time that spatial mapping of bacterial velocities has been achieved in this setting. Thus, this analysis technique provides opportunities to better understand this phenomenon and to drive it towards in vivo applications.

## 1. Introduction

Bacteria are often described as “intelligent” or as “natural microrobots” because they can autonomously swim toward tumor regions by diverse taxis mechanisms—for example, chemotaxis as a response to chemical gradients near tumors [1]. However, this autonomous motion of bacteria is slow and is limited in transport length when considering its targets (e.g. tissues, organs) due to the difference in size between bacteria (a few microns) and tissues and organs (up to several centimeters). External energy sources—such as magnetic fields, acoustic waves, and optical tweezers—have been used to enhance guidance and transport of bacteria (and of nano/microparticles and microrobots) [2–8]. Interestingly, robust and non-invasive actuation, transport and control of bacteria (and of other untethered micro-objects and microrobots) may provide numerous opportunities for clinical applications.

For example, in the field of drug delivery, controlling bacterial ‘swarms’ has been proposed as a method for targeting bacteria to specific regions in order to deliver payloads of drugs. Increasing the size of the bacterial swarms is desirable to increase both targeting efficiency and local dosage, which can lead to better treatment efficacies and outcomes [9]. However, only a limited number of studies have been reported on the formation and guidance of swarms of bacteria for enhanced targeted drug delivery. Martel et al*.* demonstrated swarming of drug-loaded magnetotactic bacteria (MTB) under an external magnetic field (which improved targeting to tumor hypoxic regions by up to 55%) in a mouse model [10]. In addition, Bhatia et al. demonstrated enhanced mass transport of nanoparticles through a microfluidic model of the vessel-tissue interface using convective flow generated by swarms of synthetic magnetic flagella and MTB [11]. Nevertheless, numerous limitations still remain in magnetic micro-manipulation of bacterial swarms: it is limited to specific bacterial species; magnetic functionalization can be required; hardware is often large; and strong magnetic fields are required [12].

Optical tweezers (OT) enable precise manipulation of micro-objects with a tightly focused laser beam [13] and can overcome some of the drawbacks described above for magnetic bacterial manipulation. Specifically, there is no limit on the species and shape of bacteria, and no extra functionalization is required, which allows more versatility in biomedical applications [14]. However, conventional OT is normally only used for manipulation of small numbers of micro-objects with precise control, as optical trapping only occurs at the tight focus of the trapping laser beam (although some applications have demonstrated multi-object manipulation using multi-trap/focus systems [15–18]). Thus, OT may not be suitable for generating swarming behavior in micro-objects. In addition, OT can be inhibited by optically absorbing or inhomogeneous (scattering) biological media (which can limit the ability to generate a focused beam for OT), particularly for in vivo applications.

An alternative mechanism for control of bacterial motion has been reported wherein optical absorption and plasmonic heating combine to generate both convection on the mm-cm scale and thermophoresis (motion of nano/microparticles in response to a temperature gradient) on the µm scale [14]. This “plasmo-thermal” effect occurs as a result of highly confined and enhanced heating caused by surface plasmon resonance (SPR) at plasmonic metal nano/microstructures [19–22]. This effect allows swarm generation while also offering flexibility in optical properties of particles/media (as penetration of light into the medium/sample is not required) and lower illumination powers relative to conventional OT. Furthermore, it does not require any complex optics (e.g. lenses, beam shaping optics, scanners, etc.) at the point of delivery [23,24]. Nevertheless, challenges lie ahead to develop more reliable, repeatable and simple methods for fabrication of plasmonic devices for bacteria swarm manipulation, particularly to enable potential in vivo applications. In addition, improved characterization of bacterial swarming in high concentration suspensions is required to allow better control of loadings and doses in bacteria-based drug delivery applications.

**Fig. 1.**
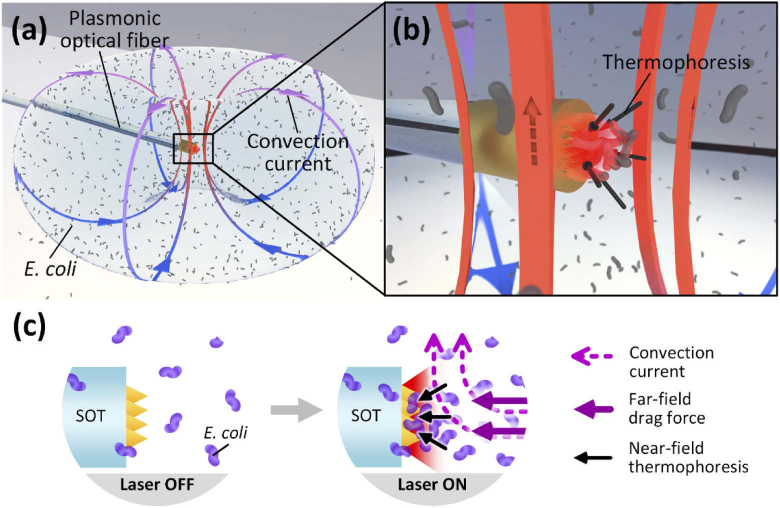
Schematic illustrations of the plasmo-thermal bacteria accumulation effect at the plasmonic optical fiber. (a) The toroidal convection current in far-field generated by highly localized plasmonic heating. (b) The zoomed in view in the vicinity of the plasmonic optical fiber tip displaying the thermophoresis-based trapping in the near-field. (c) Side view illustrations of the effect without and with the laser illumination.

Here we demonstrate high-density bacteria accumulation—based on the above plasmo-thermal effect—using plasmonic microstructures on the tips of optical fibers (Fig. 1). Importantly, the fiber-optic nature of the devices indicates the potential for future in vivo applications such as bacteria-based drug delivery. Targeted drug delivery has received much interest in recent years, particularly for cancer treatment where it has the potential to not only improve the effectiveness of chemotherapy but also to minimize side effects and complications caused by indiscriminate exposure of healthy regions [25]. Highly targeted local administration of drugs, such as via intratumoral injection, therefore, has been actively studied as a solution to overcome such limitations [26,27]. Although intratumoral injections of drugs are feasible for various cancers and target organs, lack of controllability of drug transport to the target site (e.g. leakage to surrounding areas), resulting in reduced efficacy, still remains a significant problem [27]. Plasmo-thermal swarm micro-manipulation is a good candidate to overcome this challenge, particularly for nanomedicine and/or (natural/artificial) microrobot-based drug delivery. As an example, fiber-optic devices such as the one reported here could be introduced into the body using currently available minimally-invasive tools (e.g. endoscopes, needles, catheters, etc.) and used to enhance targeting/transport of drugs to tumors. This could potentially be achieved by locally positioning the fibre-optic devices near to the target tissues (alongside the drug delivery tubes/needles) even in the cases of deep lesions or lesions within visceral organs. Plasmo-thermal trapping could then help to stop the drugs from diffusing widely and, hence, enhance drug absorption in the target tissues. Hence, fiber-optic plasmo-thermal trapping tools alongside various existing therapeutic and diagnostic interventions (such as imaging-guided interventions and optical biopsies [28–30]) present numerous opportunities to aid and/or improve in vivo, targeted drug delivery.

We recently developed a reproducible and controllable fabrication technique using two-photon polymerization (2PP) to create plasmonic arrays on both planar substrates and optical fibers for surface-enhanced Raman spectroscopy (SERS) [31,32]. The fabricated plasmonic arrays not only exhibited strong SERS effects but also showed noticeable bacteria trapping capabilities on planar substrates, where attraction of swimming *Escherichia coli* (*E. coli*) was observed in low concentration suspensions (Supplement 1 Fig. S1, Visualization 1). This effect was impacted by the geometry of the plasmonic array, and the geometry optimized for the best SERS effect [32] also exhibited the strongest attraction (i.e. the largest number of *E. coli* were attracted, Fig. S1 in Supplement 1).

In this work, we focus on this effect and comprehensively investigate the high-density bacteria accumulation performance using the plasmonic optical fiber in high concentration *E. coli* suspensions for potential enhanced bacteria-based drug delivery applications. Together with detailed video analyses, we propose a new approach to analyze the accumulation motion of bacteria across large areas in such high-density suspensions. By borrowing an analysis method from laser speckle imaging (LSI)—a well-established technique that is used widely in the biomedical optics community—we achieve 2-dimensional spatial mapping of bacterial velocities during plasmo-thermal accumulation for the first time. This presents key opportunities to improve understanding of plasmo-thermal bacterial accumulation and to better design plasmonic microstructures for this purpose. Furthermore, because the plasmonic optical fiber is thin (220 µm) and flexible, our results demonstrate the potential for in vivo applications, such as enhanced bacteria-based drug delivery.

## 2. Background

### 2.1 Plasmo-thermal bacteria trapping

Surface plasmons are collective oscillations of charges at the interfaces between metallic surfaces and dielectric media. Surface plasmons are excited by light, and SPR occurs at the resonant condition (i.e. when the momentum of the illumination light is matched with that of the plasmon) [33]. When SPR occurs, it leads to strong absorption and/or scattering of light. The absorption can then locally generate heat at the metal surface, which has been utilized for various applications including cancer therapy [20,22].

Moreover, this highly localized heating effect (confined within a few hundred nanometers of the plasmonic surface) also exhibits excellent micro-manipulation performance [34–37]. As discussed above, this plasmo-thermal effect is particularly useful for accumulation/control of swarms of micro-objects, and is driven by a unique combination of thermal convection in the far-field and thermophoresis-based trapping in the near-field (Fig. 1). The convection current is generated by spatial instability of the fluid due to the highly localized heat source and has a range of up to a few hundred micrometers [38]. The thermophoresis, on the other hand, is induced by the temperature gradient in the vicinity of the plasmonic heat source, and acts on the bacteria with a range of approximately 10 μm. The particle flux due to diffusion ($\vec{J}$)—which includes thermophoretic motion—is given by

(1) $$\vec{J}\;=-D\nabla c-cD_{\text{T}}\nabla T$$

where *D* is the Fickian diffusion coefficient, *c* is the particle concentration (number of particles per unit volume), *D*_T_ is the thermal diffusion coefficient, and *T* is the temperature [39]. The second term on the right-hand side of Eq. (1) represents the particle flux due to the temperature gradient, and thus the thermophoretic transport. The ratio *S*_T_ = *D*_T_/*D* is referred to as the Soret coefficient and indicates the strength and direction of thermophoresis—particles migrate from warmer regions to cooler regions when *S*_T_ > 0, and vice versa.

Thermophoresis is a sophisticated phenomenon influenced by various factors (e.g. temperature, pH, ion concentration, solvent expansivity, particle shape and surface chemistry) that is yet to be fully understood [39–41]. Nonetheless, negative *S*_T_ values—demonstrating accumulation at warmer regions—have been reported for microparticles and bacterial (and other) cells in aqueous solutions in several recent studies [37,42–45], including a small number of investigations using fiber-optic devices [24,46,47]. The fabrication of such fiber-optic devices for thermophoretic manipulation has been achieved using a variety of techniques including coating optical fiber tips with graphene [46] and depositing gold thin films and annealing to create random nano-islands on either flat fiber tips or tapered fiber tips [24,47]. While these techniques are effective for fabrication of plasmonic structures on optical fiber tips, they suffer from a lack of controllability and repeatability. In this work, we fabricated our plasmonic optical fiber using 2PP, thereby facilitating direct 3D printing of arbitrary nano/microstructures onto fiber tips. Crucially, this fabrication process is highly reliable, repeatable and controllable which allows us to optimize and tailor the manipulation performance. To the best of our knowledge, this is the first report demonstrating plasmo-thermal effects and manipulation using 2PP-fabricated plasmonic optical fibers.

### 2.2 Laser speckle imaging

Laser speckle imaging (LSI) is an imaging technique that has been widely used in biomedical research to map blood flow [48,49]. By assessing fluctuations in a speckle pattern generated by a coherent light source, it is possible to quantify the rate and direction of flow within the sample, as the flow patterns produce transient changes in the shape of the sample surface (which in turn effect changes in the speckle pattern) [48]. This has been most widely applied to blood flow imaging [50,51] but can also be used for imaging of other fluids/particles.

To quantify flow rates, speckle contrast values are calculated (at each image pixel) as the ratio of the standard deviation of the image intensity to the average image intensity. The average and standard deviation values can be calculated spatially (i.e. across a number of image pixels) or temporally (i.e. over a series of consecutive image frames). The speckle contrast, *K*, is thus defined as

(2) $$K=\frac{\sigma_{i}}{\left\langle I\right\rangle }$$

where 〈*I*〉 and *σ_i_* respectively represent the mean intensity and standard deviation.

The speckle contrast is proportional to the speckle decorrelation time, *τ*_c_, which represents the time taken for the speckle image to completely decorrelate from a starting image. In turn, the speckle decorrelation time is inversely proportional to the flow velocity, *V* (as shown in Eq. (3) below), as higher flow rates generate more rapid changes in speckle patterns and, hence, produce shorter decorrelation times and lower speckle contrast values.

(3) $$V\propto \tau_{c}^{-1}\propto K^{-1}$$

In the experiments presented here, we observed motion of bacteria under an optical microscope with white light (incoherent) illumination (i.e. no laser illumination was used for the purpose of imaging). As the white light image resolution (pixel size) was comparable to the size of individual bacterial cells, movement of the bacteria induced fluctuations in the brightness of image pixels in a ‘speckle-like’ manner. For this we reason, we chose to apply speckle image analysis to the bacterial motion images. In this way, we were first able to obtain speckle contrast maps in which the pixel values were proportional to bacterial flow rates. By combining this analysis with manual frame-by-frame tracking of bacteria—in which we quantified the velocity—we were then able to generate a calibration curve to convert speckle contrast values into bacterial velocities. As such, this approach allowed us to quantify and spatially map the velocities of bacteria under plasmo-thermal trapping.

It is worth noting that there are other methods available to visualize nano/microparticles under plasmo-thermal trapping, including particle tracking analysis based on mean square displacement (MSD) calculation [52]. Nevertheless, most current methods rely on identifying and tracking the trajectory of individual particles from frame to frame. This is not only time demanding but is also less versatile/practical as it is applicable to a limited number of particles in a discrete manner. Conversely, the speckle imaging technique used here does not require individual particle identification and tracking after the initial calibration and provides analysis of changes in every image pixel. Thus, it is more time efficient and less computationally intensive than particle tracking techniques while also allowing simultaneous analysis of motion occurring across large areas.

## 3. Methods

### 3.1 Plasmonic optical fiber fabrication

To fabricate plasmonic structures on the tips of optical fibers, low-OH multimode silica optical fibers (200/220 µm core/cladding diameter, 0.22 NA, FG200LEA, Thorlabs, Inc., Germany) were first cut into 30 cm long sections using an automatic fiber cleaver (CT-101, Fujikura Ltd., Japan). The cleaved fibers were then clamped on a 3D-printed holder as described in the previous work [32].

**Fig. 2.**
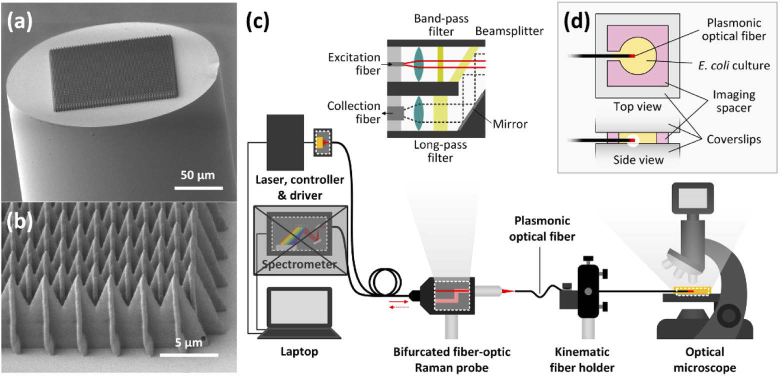
The fabricated plasmonic optical fiber images and experimental setup. (a,b) Scanning electron microscope (SEM) images of the tip of a plasmonic optical fiber and details of the plasmonic structure array. (c) Experimental setup. One arm of the proximal end of the bifurcated fiber-optic Raman probe is connected to a spectrometer for spectral data collection. However, the spectrometer is masked with a gray box for clarity as it was not used in this work. (d) Detailed illustration of the imaging chamber.

A Photonic Professional GT system (Nanoscribe GmbH., Germany) was used for 2PP on the tips of the optical fibers (Fig. S2 in Supplement 1). A droplet of photoresist (IP-Dip, Nanoscribe GmbH., Germany) was cast on a 63× immersion objective lens (NA = 1.4; ZEISS, Germany) and then the 3D-printed fiber clamping holder was placed on the system sample holder. The lens height was then raised until the tip of the optical fiber was immersed in the droplet. Afterwards, the interface between the end face of the optical fiber and the photoresist was carefully found by finely adjusting the position of the microscope objective. Once the interface was found, the 2PP process was launched. In this study, we adhered to the microstructure array design (total area of 125 × 125 μm^2^) for which we observed the highest SERS enhancement (on plasmonic optical fiber probes) in previous work (cross spike array (CSA, Fig. 2(a),(b)) with 4 μm width, 5 μm height, and 2.8 μm spacing distance) [32]. The 2PP laser (*λ* = 780 nm) exposure parameters were 5 mW and 1 ms for the laser power and exposure time respectively.

Once 2PP was complete, the microstructure arrays were developed by soaking the tips of fibers in propylene glycol methyl ether acetate (PGMEA) for 20 minutes and then rinsing the residual photoresist using isopropyl alcohol (IPA). Finally, the optical fibers were placed in a metal sputtering chamber (HEX modular deposition system, Korvus Technology, UK) in order to deposit a gold thin film (50 nm thickness) on the 2PP structures under vacuum.

### 3.2 E. coli sample preparation

*E. coli* K12 J53-1 (ATCC BAA-769, ATCC, USA) were cultured in autoclaved tryptic soy broth (Sigma-Aldrich, Germany) at 37℃ overnight and purified for use in bacterial accumulation experiments. For purification, 10 ml of the saturated culture was centrifuged (3 min, 4000 rpm, MiniSpin, Eppendorf, Germany) and the pellet was re-suspended and rinsed three times with phosphate buffered saline (PBS). The resulting suspension was then used for the subsequent imaging experiments, with 1 ml samples used in each case.

### 3.3 Imaging of E. coli accumulation/diffusion

The experimental setup used for imaging of *E. coli* accumulation included a laser diode (785 nm continuous wave, L785P090, Thorlabs, Inc., Germany), a controller (LDM9 T/M, Thorlabs, Inc., Germany), a laser diode driver (LDC202C, Thorlabs, Inc., Germany), a spectrometer (QE Pro, Ocean Optics, Inc., the Netherlands), a commercial bifurcated fiber-optic Raman probe (InPhotonics RIP-RPB-785-FC-SMA, Ocean Optics, Inc., the Netherlands), and a compact microscope (LCD Digital Microscope II, Celestron, LLC., USA) (Fig. 2(c)). The proximal end of the plasmonic optical fiber was clamped and aligned to the Raman probe using a kinematic fiber holder to allow coupling of illumination light into the plasmonic optical fiber. The illumination laser power at the tip of a 30 cm long optical fiber was 4.7 mW.

An imaging chamber was created by stacking three imaging spacers (9 mm chamber diameter and 0.12 mm thickness for each, SecureSeal Imaging Spacer, Grace Bio-Labs, USA) on a coverslip. An opening was manually cut into one edge of the imaging chamber using a scalpel to allow the plasmonic optical fiber to be translated through the opening and into the chamber (Fig. 2(d)). Once prepared, 1 ml of *E. coli* suspension was pipetted into the imaging chamber, which was then covered with another coverslip to secure the *E. coli* suspension. The sandwiched coverslips containing the *E. coli* suspension were then placed on the microscope. Once the focus of the objective (10×; the camera sensor acts as 10× eyepiece lens, thus yielding 100× magnification power) was set within the suspension, the distal end of the plasmonic optical fiber was carefully inserted into the *E. coli* suspension through the opening in the imaging chamber. Finally, video imaging (30 frames/second) of bacteria accumulation and diffusion at the tip of the plasmonic optical fiber was recorded under the microscope with transmitted illumination.

### 3.4 Video analysis

The recorded video clips were analyzed using MATLAB. First, the spatial and temporal bacteria concentration changes were examined by extracting the average pixel intensities (*I*) of twenty 10 × 10 μm^2^ square regions of interest (ROIs) over all frames in five different directions (i.e. left-horizontal, left-diagonal, vertical, right-diagonal, and right-horizontal). When extracting pixel intensity (*I*) values, the brightness and contrast of all frames were adjusted to maximize bacterial contrast, and the frames were converted to 8-bit grayscale images (with corresponding pixel value ranges from 0 to 255). Importantly, the brightness and contrast adjustments applied were the same for all frames, and this process involved re-scaling the pixel intensity histogram ranges (from 0-198.9 to 25.5-255, Fig. S3 in Supplement 1). Due to the transmitted illumination imaging configuration, bacteria cells either block or scatter the imaging light, and therefore, the pixels in which bacteria are observed exhibit lower *I* values. As a result, the darker the grayscale in a given ROI, the higher the bacteria concentration. Accordingly, an arbitrary concentration value, *C* (a parameter representing the relative bacteria concentration), was defined as *C* = 255 – *I*.

Secondly, the distances of bacteria cells from the center of the fiber tip were measured over consecutive image frames to calculate the in-plane velocity of the bacteria. Bacteria cells were randomly chosen (19 cells in total) and for each cell the change in the distance between the center of the fiber tip and the center of mass of the area occupied by individual bacteria cells (*r*, manually identified in each frame) was measured over 30 consecutive frames (for 1 sec) from when the laser was switched on. All graphing and curve fitting for analysis of bacterial concentrations and velocities were carried out using Origin 2020.

### 3.5 Speckle imaging analysis

To obtain velocity mapping across the whole area of the images, the temporal speckle contrast, *K*, was computed across consecutive frames. Firstly, the raw video frames were converted to grayscale images and then their histograms were adjusted to match the histogram of a chosen reference image. The selected reference frame was taken from the beginning of the acquisition before any background brightness changes occurred, and this process thus eliminated errors due to this effect.

Once the image pre-processing was complete, a time window (*t*_w_) was determined for calculation of the speckle contrast and the appropriate frames were extracted for analysis. Subsequently, the mean intensity (⟨I⟩) and standard deviation (*σ_i_*) of each pixel were computed across the frames within *t*_w_ to obtain the *K* (speckle contrast) image. Finally, velocity mapping was achieved by calculating the inverse *K* image (*K*^-1^) and by correlating the inverse *K* profiles with the frame-by-frame velocity measurement plot. For the speckle analysis, the tip of the plasmonic optical fiber was filtered out of the images as the pixels in that area were not relevant to the bacterial motion.

## 4. Results and discussion

### 4.1 Variation in spatial and temporal bacteria concentration under plasmo-thermal trapping

**Fig. 3.**
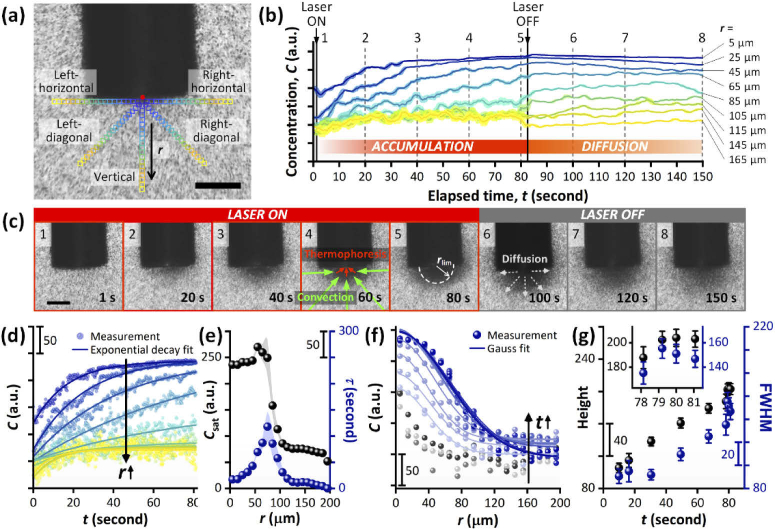
Characterization of spatial and temporal variations in bacterial concentration. (a) Optical microscope image of plasmonic optical fiber probe in *E. coli* solution (the frame before the laser was switched on). 20 square regions of interest (ROIs; colored squares; individual ROI area = 10 × 10 μm^2^) were generated in five different directions from the center of the tip (red dot). Scale bar: 100 μm. (b) Moving averaged concentration change over time at selected ROIs in the vertical direction (at distances, *r* = 5/25/45/65/85/105/115/145/165 μm). The shaded areas represent the standard deviations of the moving averaged concentration profiles. For each time point, the moving average was calculated as the average of the concentration values at the current time point and the five time points either side. (c) Frames at 1/20/40/60/80/100/120/150 s corresponding to the dashed lines in (b). Scale bar: 100 μm. (d) Exponential decay fits to the selected *C*-*t* plots during the accumulation phase (at *r* = 5/25/45/65/85/105/115/145/165 μm). Major scale tick: 50 a.u. (e) Saturation concentration (*C*_sat_, black) and time constant (*τ*, blue) with respect to distance from the center of the fiber tip (*r*), obtained from the exponential decay fits. (f) Gaussian fits to ten representative *C*-*r* plots during the accumulation phase (at *t* = 10/16/30/49/67/77/78/79/80/81 s). Three early stage plots are displayed in gray shades (*t* = 0/1.6/4 s) to demonstrate the difference in shape between early and developed *C*-*r* plots. Major scale tick: 50 a.u. (g) Height (black) and FWHM (blue) of the Gaussian curves with respect to elapsed time (*t*). Inset: the zoomed plot of data points in the time range from 78 s to 81 s, which demonstrates that the curves may have reached plateaus.

To investigate plasmo-thermal bacteria accumulation at the tip of plasmonic optical fibers (Fig. 2(a),(b)), we imaged the bacterial motion induced by the fibers in custom imaging chambers under an optical microscope with incoherent, white light illumination (Fig. 2(c),(d); further details in section 3.3 Imaging of *E. coli* accumulation/diffusion). Results on the characterization and analysis of the spatial and temporal bacteria concentration variations are displayed in Fig. 3. First, the local concentration changes over time along five different directions were extracted from white light video data as *E. coli* were observed to accumulate at the tip of a plasmonic optical fiber delivering 785 nm laser illumination (Fig. 3(a),(b), Visualization 2). Once the laser was switched off, the accumulation behavior ceased, and the bacteria were observed to slowly diffuse away from the fiber tip (Fig. 3(b),(c), Visualization 2). The local concentration changes over time were similar in the five directions ( Fig. S4, Supplement 1), which indicated homogeneous *E. coli* accumulation and diffusion behavior across the area around the fiber tip.

Despite this homogeneity, as the tip of the plasmonic optical fiber placed a geometrical restriction on the bacterial motion along the horizontal and diagonal directions, the concentration versus elapsed time plots in the vertical direction were chosen for further analysis. A prompt response to the laser exposure was observed in both accumulation (laser on) and diffusion (laser off) phases (Fig. 3(b), Fig. S5, Supplement 1). Importantly, the attractive motion—and thus the resulting concentration increase—were observed across the entire area of the imaging frame (W570 μm × H427.5 μm, Fig. 3(c); W1300 μm × H975 μm with a lower magnification, Visualization 3). This indicated a strong far-field effect attracting bacteria located far away from the plasmonic optical fiber.

This far-field effect was attributed to a convection current associated with a highly localized plasmonic heating created by the plasmonic array at the fiber tip. A toroidal current centered at the plasmonic array was generated, as demonstrated by a reversal of the flow direction as the depth of the focal plane was shifted (see Visualization 4). One might typically refer to this toroidal current as traditional Rayleigh-Bérnard convection. However, the mechanism of the plasmo-thermal convection should be treated differently. This is because the onset of buoyancy-driven natural convection is initiated by instability due to a temperature gradient in a system having a uniform heat source (e.g. infinite walls and planes), whereas the plasmo-thermal convection is triggered by spatial instability due to a highly localized heat source [38]. To demonstrate this, a dimensionless fluid dynamic parameter, the Rayleigh number (Ra), can be considered. Ra is the ratio of the time scale for diffusive thermal transport to the time scale for convective thermal transport and is written as

(4) $$Ra=\frac{\rho \beta l^{3}g\Delta T}{\mu \alpha }$$

where *ρ* is the density, *β* is the volumetric thermal expansion coefficient, *l* is the characteristic length of the heat source, *g* is the gravitational acceleration, Δ*T* is the temperature difference between the surface of the heat source and the ambient (far away from the heat source), *μ* is the dynamic viscosity, and *α* is the thermal diffusivity of the fluid.

The critical value of Ra, which determines which thermal transport mechanism is dominant, is 1700—if Ra of the system exceeds this value, natural convection occurs, otherwise the system is quiescent and heat transfer is by diffusion (conduction) [53]. When values for water at room temperature (*ρ*_w,20_ = 997 kg/m^3^, *β*_w,20_ = 0.18×10^−3^ K^-1^, *μ*_w,20_ = 10^−3^ Pa·s, *α*_w,20_ = 0.143 × 10 ^−6^  m^2^ /s) and the footprint of the plasmonic array at the tip (*l* = 1.25×10^−4^ m) are applied, it indicates that the system requires Δ*T* > ∼70,000℃ to generate a convective flow (i.e. meeting Ra > 1700). Aside from the fact that evaporation was not observed (indicating that temperatures above 100℃ were highly unlikely to have been generated), it was also reported that the maximum temperature that can be achieved through super-heating of plasmonic nanoparticles was lower than 240℃ [54]. Accordingly, this confirms that the plasmo-thermal convection current is not likely created by traditional Rayleigh-Bérnard convection (as the temperature generated at the plasmonic array is not high enough). Instead, the temperature profile at the plasmonic array induces the desired instability (and, hence, induces convection) at any positive Δ*T* (Ra > 0) due to the highly localized nature of the plasmonic heating [38].

Importantly, bacterial cells were not observed to be damaged or negatively impacted by the plasmonic heat source, which was attributed to the surrounding aqueous medium having a low thermal diffusivity (hard to raise the temperature due to a combination of the thermal conductivity and the specific heat). Indeed, observations on planar substrates (Fig. S1 in Supplement 1, and Visualization 1) showed no noticeable heat damage to the bacteria. Consequently, most scenarios with biological samples (which are typically surrounded by aqueous environments) would not experience negative thermal effects in plasmo-thermal accumulation, thus suggesting that this technique may be suitable for in vivo applications (such as drug delivery) in the future.

### 4.2 Spatial limit to bacteria accumulation

There was a limit of the distance where the *E. coli* accumulated. This became more evident by fitting the concentration variation plots with curves as functions of time and distance. First, good fits were achieved for the concentration vs. time (*C*-*t)* plots using exponential decay functions expressed as *C*(*t*) = *C*_sat_ + *a* exp(-*t*/*τ*), where *C*_sat_, *τ*, and *a* are the maximum saturation concentration, the time constant, and a coefficient related to the initial concentration, respectively (average R^2^ = 0.900, Fig. 3(d)).

This meant that the bacterial concentration increased over time until a maximum concentration (*C*_sat_) was reached. The further from the center of the tip, the longer the time required to reach *C*_sat_, up until a certain distance. However, after a certain distance (*r*_lim_ = 75 μm), the saturation point was reached more quickly and the *C*_sat_ value was very low. This indicated that an equilibrium state had been reached and thus there was no effective accumulation (Fig. 3(e)).

Further cross-confirmation was carried out by fitting the concentration vs. distance (*C*-*r)* plots with Gaussian curves. By considering the peak concentration position at *r* = 0, Gaussian curves were fitted for ten representative plots (average R^2^ = 0.969, Fig. 3(f)). The heights and the full width at half maximum (FWHM) values of the curves were then plotted with respect to the elapsed time (*t*) to confirm the limited effective accumulation range and the time when the accumulation appeared to be fully matured (Fig. 3(g)). The FWHM of the fully matured accumulation effect was attained after 79 seconds and was ∼150 μm, which corresponded to the *r*_lim_ value obtained earlier (i.e. 2 × *r*_lim_ = 2 × 75 μm = 150 μm). This value is also reasonable considering that the dimensions of the plasmonic array were 125 × 125 μm^2^. As a result, this suggested that the near-field thermophoretic force applied selectively in the vicinity of the plasmonic array (i.e. within 150 μm). However, it is worth noting that the data shown in Fig. 3(g) contains a limited number of timepoints after the time at which the fully matured accumulation was observed. As such, data over longer time periods would be beneficial in further confirming the above observation.

### 4.3 Thermophoretic force calculation

**Fig. 4.**
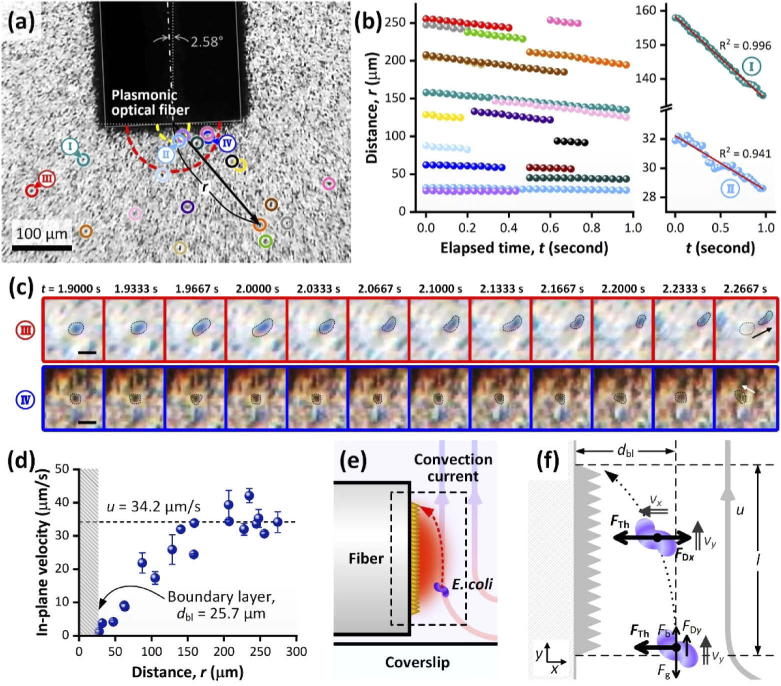
Frame-by-frame tracking of bacteria cells and bacteria trapping force calculation. (a) An initial frame displaying the center of the tip—which is the reference point to measure the distance (*r*) to bacteria cells—and the 19 tracked bacteria cells. Zero in-plane velocity region (boundary layer) is shown within a distance of *r* = 25.7 μm (yellow dashed line) along with the matured swarm size limit (*r*_lim_ = 75 μm, red dashed line). (b) Distance versus time plots for the 19 bacteria cells indicated in (a) and two examples of linear fitting on the plots (positions I and II displayed in (a)).The plots in (b) are color coded with respect to the labelling colors of the selected bacteria cells in (a). (c) Example images showing manually tracked bacterial motion over the first 12 frames during the accumulation (zoomed into two bacteria cells; positions III and IV displayed in (a)). Scale bars: 10 μm. (d) In-plane velocity (scalar) versus distance plot obtained from the linear fits of each plot in (b). The error bars are the standard errors of the slopes of the linear fits. The range of the boundary layer where the bacteria are stationary in plane is highlighted in gray. (e) A schematic diagram of single bacterium trapping scenario near the plasmonic optical fiber tip. (f) The force and motion diagram applying to the body of a bacterium at two locations in the vicinity of the fiber tip.

To further analyze the *E. coli* accumulation effect, the in-plane velocity of bacteria was measured by tracking cells’ locations frame-by-frame for the first 30 frames (1 s) after the laser was switched on (Fig. 4(a)). The variations in the locations were linear with respect to time, which indicates that bacterial velocities were approximately constant over the 1 s timeframe (Fig. 4(b)). Importantly, the transmitted illumination configuration offered a high enough image contrast and resolution to identify individual cells, and measurements for the frames at the early stage of the accumulation—in which the concentration near the tip was still low—permitted manual tracking (Fig. 4(c)). Moreover, although the sizes of the *E. coli* cells (typically, 1 μm in width × 2 μm in length) were comparable to the pixel size (approx. 0.89 × 0.89 μm^2^), the in-plane velocity was calculated over a path segment corresponding to a range of 31.2 to 308 pixels (27.8 to 274 μm). Linear fitting of the *r-t* curves was used to calculate bacterial cell velocities, and over this range (27.8 to 274 μm) the average standard error on the fits was found to be <10%.

When plotting the measured *E. coli* velocities at different locations, it appeared that the velocity increased with respect to distance away from the tip until a threshold was reached at 34.2 μm/s (at approximately 200 μm from the fiber tip; see Fig. 4(d)). In addition, the velocity dropped to zero at distances below 25.7 μm from the fiber tip, which suggested a boundary layer at the tip of the plasmonic optical fiber (highlighted in gray in Fig. 4(d)). This reduction in velocity close to the fiber tip was attributed to the flow direction changing along the depth direction due to the toroidal convection current (i.e. close to the fiber tip, the convection current pushes bacteria upwards rather than towards or away from the fiber). In turn, this effect acted to reduce the in-plane velocity toward the plasmonic optical fiber.

The maximum in-plane velocity measured by tracking the bacteria (34.2 μm/s) corresponded to the plasmo-thermal convection current flow velocity (*u*) as the cells are small enough to act as perfect tracers. For the laminar flow condition (Reynolds number ≪ 1, which our case meets—see Supplement 1, Section 1), the Stokes number (St; the parameter to determine whether particles in fluid follow the flow or do not when the flow direction is changing due to obstacles, etc.) is defined as St = *ρ*_p_*d*_p_^2^*u*/18*μ*_f_*L*. In this equation, *ρ*_p_ is the particle density (mass/volume), *d*_p_ is the particle diameter, *u* is the flow velocity, *μ*_f_ is the fluid dynamic viscosity, and *L* is the characteristic length of the flow/obstacle. In our case, *ρ*_p_ = *ρ_E.coli_* = 1.105×10^3^ kg/m^3^, *d*_p_ = *d_E.coli_* = 1.075 μm (as the volume equivalent diameter), *u* = 34.2 μm/s, *μ*_f_ = *μ*_w,20_ = 10^−3^ Pa·s, and *L* = 220 μm as the diameter of the fiber. These values gave St ≪ 1, which confirmed that the particles should follow the convection current streamline even though the plasmonic optical fiber acted as an obstacle to the flow.

Despite this, *E. coli* cells were observed to accumulate at the fiber tip, implying that an additional ‘trapping’ force must be acting on the bacteria. Having obtained the flow velocity, this thermophoretic force attracting the bacteria to the fiber tip could be estimated according to the situation illustrated in Fig. 4(e,f). We considered a single bacterium in fluid, traveling along the convection current flow at the border of the boundary layer (*d*_bl_ = 25.7 μm). Since the bacterium perfectly follows the flow stream (*v_y_* = *u*), the gravitational force (*F*_g_), the buoyant force (*F*_b_), and the *y*-directional drag force (*F*_D*y*_) balance out so that there is no net force in the *y*-direction (Fig. 4(f)). When the bacterium enters the vicinity of the plasmonic array, it starts to experience the thermophoretic attraction force in the negative *x*-direction (*F*_Th_). While the bacterium travels above the plasmonic array at constant velocity, *v_y_*, the negative *x*-directional acceleration due to *F*_Th_ induces a velocity in the negative *x*-direction (*v_x_*) towards the fiber tip. As a result, a drag force (*F*_D_) in the opposite direction starts to apply.

By solving the motion equations for this system (a detailed step-by-step calculation is presented in Supplement 1, Section 2), an acceleration of −1.91 μm/s^2^ and thus a maximum velocity (*v_x_*) of −9.91 μm/s were obtained for the bacterium being trapped. Here the negative sign indicates that the direction of the thermophoretic trapping force (and the motion) is toward the fiber tip (Fig. 4(f)). The drag force is defined by Stokes’ law as *F*_D_* *= −3*πμ*_w_*d*_p_*v_x_* = 100 fN. Thus, to securely trap the bacterium, the magnitude of *F*_Th_ should be greater than that of *F*_D_, i.e. |*F*_Th_| > 100 fN. This value is well aligned with the magnitude of thermophoretic forces reported in the literature (at the relevant scale), which range from 20 to 100 fN [42,55].

### 4.4 Speckle imaging analysis

**Fig. 5.**
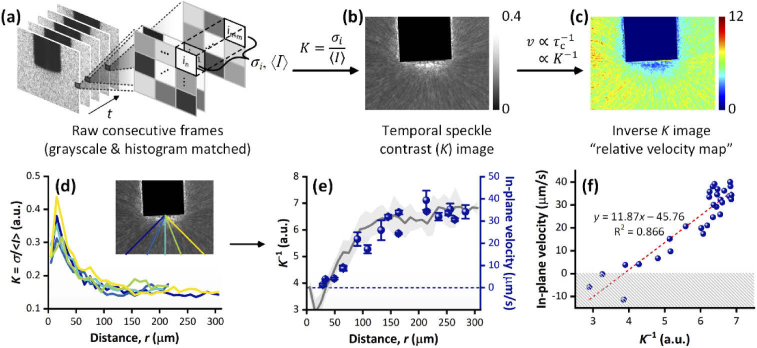
Speckle imaging analysis on the *E. coli* accumulation at the tip of the plasmonic optical fiber. (a)-(c) The process of temporal speckle imaging analysis on the recorded white light video data. The time window for analysis was set to 15 seconds and the fiber in the image was filtered out (i.e. set to zero). (d) Temporal speckle contrast (*K*) profiles along five directions from the center of the fiber tip (inset). (e) Averaged *K*^−1^ profile (gray line; light gray area represents the standard deviation) alongside the in-plane velocity measured by frame-by-frame tracking (blue scatter plot; error bars are the standard errors of the slopes of the linear fits of *r*-*t* plots in Fig. 4(b)). (f) In-plane velocity vs. *K*^−1^ plot, where the in-plane velocity values were interpolated for the full distance range (5 to 305 μm) to obtain in-plane velocity and *K*^-1^ values that share the same distance. The red dashed line represents the linear regression. The hatched area indicates the negative velocity amplitude region, which was mathematically sound but not physically meaningful. Thus, *K*^−1^ lower than 3.855 (*K*^−1^ at *v* = 0 μm/s) was regarded as zero velocity.

Finally, speckle image analysis was applied in order to map the bacteria in-plane velocity (magnitude) over the entire area of the imaging frame. Figure 5(a)-(c) present the overall image processing protocol and the intermediary-step images of speckle image analysis on the bacteria accumulation video data. The temporal speckle contrast, *K*, was calculated at each pixel over consecutive frames corresponding to a chosen time length (*t*_w_). The *K* values and the corresponding inverse *K* values across the entire area of the imaging frame were then visualized in pseudo-color maps, as shown in Fig. 5(b),(c) respectively.

It should be noted that choosing the right time window (*t*_w_) was crucial because it determined the resolution and the sensitivity of the data. Indeed, *K* images with *t*_w_ larger than 20 s exhibited clear losses in spatial resolution (as observed in Fig. S6 in Supplement 1 where images are observed to become less pixelated and more blurred as *t*_w_ increases). On the other hand, using too small a *t*_w_ value resulted in noisy profiles with substantial errors (Fig. S7, Supplement 1). Consequently, the optimal *t*_w_ value was selected as 15 s.

To correlate *K*^−1^ values with actual bacteria velocities, *K* vs. distance (*r*) profiles were extracted in five directions (Fig. 5(d)). The inverse of the average *K*-*r* profile was then used for correlation with the actual in-plane velocity profile obtained in the previous section (Fig. 5(e)). Notably, the in-plane velocity profile correlated well with the *K*^−1^ profile, which suggested a linear relationship between the two. Indeed, a linear correlation between in-plane velocity and *K*^−1^ was confirmed by an interpolated in-plane velocity vs. *K*^−1^ plot (Fig. 5(f)) (due to the discrete data points in both the in-plane velocity and *K*^−1^ data, the in-plane velocity values in Fig. 5(f) were calculated using interpolation in order to obtain velocity and *K*^−1^ values that shared the same distance).

**Fig. 6.**
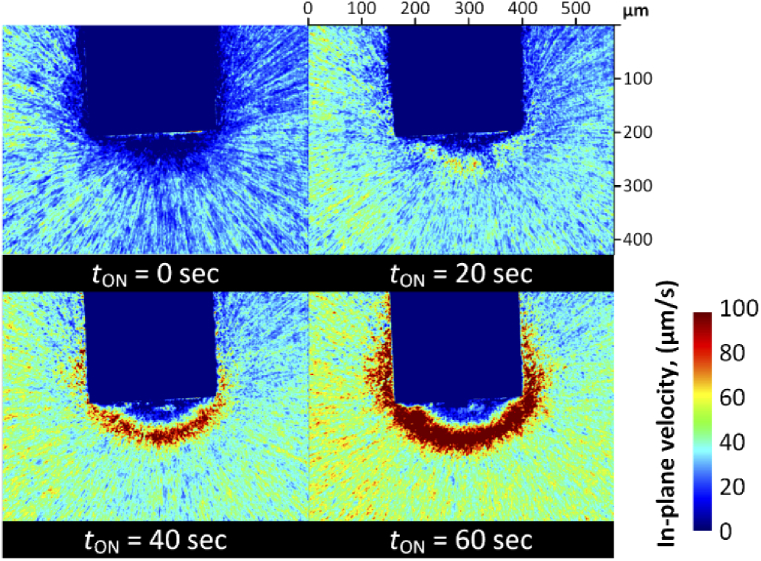
Velocity mapping results at different time frames. The parameter *t*_ON_ represents the time after the laser was switched on. The velocity mapping images were obtained by processing the frames in time windows from *t*_ON_ to *t*_ON_ + 15 s.

From the linear relationship between velocity and *K*^−1^, the *K*^−1^ images were simply converted into in-plane velocity map images (Fig. 6). Decreasing velocity toward the plasmonic optical fiber tip, as also measured by frame-by-frame tracking, appeared across the entire area of the frame. Furthermore, owing to the spatial resolution of the images, visual discrimination of the moving particles (*E. coli*, higher velocities, cyan to yellow pseudo-colors) from the background (zero velocity, dark blue pseudo-color) was possible. Thus, the bacterial motion trajectories could be clearly visualized.

Figure 6 shows velocity (*K*^−1^) images at four different time points during bacterial accumulation (*t*_ON_ = 0, 20, 40, and 60 s, where *t*_ON_ is the time after the laser was switched on). This allowed analysis of the time-dependence of the bacterial accumulation behavior. First, the overall bacterial in-plane velocity increased over time. This was attributed to an increase in the temperature gradient in the vicinity of the plasmonic array over time, as the thermophoretic drift velocity is proportional to the temperature gradient (*v*_T_ = −*D*_T_∇*T*, where *D*_T_ is the thermal diffusion coefficient) [40]. We note here that, due to the highly localized heating and the low thermal diffusivity of the surrounding medium, there is a considerable mismatch in time constants between plasmonic structures in dry states (< 1 ms) and submerged in fluids (approx. 40 s) [56]. This can lead to an increase in the local temperature gradient over time, as observed here.

It should also be noted that as the accumulation region grew at the tip of the plasmonic optical fiber (Fig. 3(f,g)), a high velocity ring emerged around the fiber tip (see images at *t*_ON_ = 40 s and 60 s in Fig. 6). This represented the boundary of the accumulated cluster and its expanding motion rather than the bacterial velocity. This anomaly occurred because there were no more speckles detected in the area of the highest bacteria concentration (in which the pixel intensity reached zero). Hence, this suggested that while speckle imaging analysis permitted wide-area spatial mapping of bacterial velocities under plasmo-thermal trapping, it may not be ideal for scenarios where there are large concentration differences. However, as this effect is linked to the camera sensor’s performance (e.g. detection range, sensitivity, etc.), it may be possible to overcome this limitation by improving the measurement settings (e.g. by using better imaging devices and auxiliary optics, such as filters, polarizers, etc.), the acquisition parameters, and the image correction parameters (i.e. brightness, contrast, etc.).

## 5. Conclusion

Bacteria can potentially be employed as natural microrobots for targeted drug delivery owing to their autonomous behavior in response to the surrounding environment. To enhance the efficiency and effectiveness of delivery, external guides to concentrate bacteria are required. Here we have demonstrated effective plasmo-thermal bacterial accumulation using a plasmonic optical fiber (fabricated via 2PP and gold sputtering) and proposed a new approach to visualize the effect over large areas by applying a speckle-based imaging analysis technique.

SPR was excited at the plasmonic array using low power (4.7 mW) illumination with a 785 nm laser source. This resulted in highly localized heating at the tip of the fiber. The large-scale *E. coli* accumulation (swarm size of approx. 150 μm) was facilitated by the combined action of convection to pull bacteria towards the tip (far-field effect) and thermophoresis to trap bacteria in the near-field. The maximum in-plane velocity was measured as 34.2 μm/s, and the estimated thermophoretic trapping force was 100 fN, which was in agreement with previous reports.

We were also able to visualize in-plane bacterial velocities over large areas—demonstrating the uniformity of the accumulation effect—by applying speckle imaging analysis to the video data. To the best of our knowledge, this is the first time that 2D spatial mapping of bacteria velocity has been achieved in this setting. Only a small number of studies have reported laser speckle imaging analysis for measurement/detection of bacterial motion, all of which involved monitoring long-term bacterial behavior (over several hours) in response to changes in the surrounding environment (e.g. for studies of colony growth, antibacterial susceptibility, etc.) [57–62]. Furthermore, our approach uses the white light images taken using a general optical microscope unlike the abovementioned examples (which all require laser illumination), thereby offering greater versatility and potential for wider applications. Hence, this speckle-based analysis technique may provide opportunities to better understand plasmo-thermal trapping and to guide design of improved plasmonic microstructures and tools for this purpose. Finally, the plasmonic optical fiber used in these experiments is thin (220 μm in diameter), flexible, and can be as long as required, thus indicating the potential for in vivo, minimally-invasive applications.

## Data Availability

Data underlying the results presented in this paper are not publicly available at this time but may be obtained from the authors upon reasonable request.

## Supplemental document

See Supplement 1 for supporting content.
