# Supplementary material for: Plasmonic optical fiber for bacteria manipulation—characterization and visualization of accumulation behavior under plasmo-thermal trapping
Source: Biomed Opt Express. 2021 Jun 8;12(7):3917–33. doi: 10.1364/BOE.425405 (PMC8367256; doi:10.1364/BOE.425405)
Supplement: Supplementary file 1 [file boe-12-7-3917-s001.pdf]

Supplemental Document

Biomedical Optics EXPRESS

Plasmonic optical fiber for bacteria manipulation—characterization and visualization of accumulation behavior under plasmo-thermal trapping: supplement

JANG AH KIM,[1,4] ERIC M. YEATMAN,[2] AND ALEX J. THOMPSON[1,3,5]

[1]*The Hamlyn Centre, Institute of Global Health Innovation (IGHI), Imperial College London, Exhibition Road, South Kensington, London SW7 2AZ, UK*
[2]*Department of Electrical and Electronic Engineering, Imperial College London, Exhibition Road, South Kensington, London SW7 2AZ, UK*
[3]*Surgical Innovation Centre (Paterson Building), Department of Surgery & Cancer, St Mary's Hospital, Imperial College London, South Wharf Road, London W2 1NY, UK*
[4]*j.a.kim@imperial.ac.uk*
[5]*alex.thompson08@imperial.ac.uk*

This supplement published with The Optical Society on 8 June 2021 by The Authors under the terms of the Creative Commons Attribution 4.0 License in the format provided by the authors and unedited. Further distribution of this work must maintain attribution to the author(s) and the published article's title, journal citation, and DOI.

Supplement DOI: https://doi.org/10.6084/m9.figshare.14625669

Parent Article DOI: https://doi.org/10.1364/BOE.425405

Plasmonic optical fiber for bacteria manipulation—characterization and visualization of accumulation behavior under plasmo-thermal trapping

This supplementary file contains:

1. Reynolds number calculation
2. Solution of motion equations for calculation of the thermophoretic trapping force
3. Descriptions of supplementary videos
4. Supplementary figures
5. References

1. Reynolds number calculation

The Reynolds number (Re) is a dimensionless number that characterizes fluidic behavior by the ratio of inertial forces to viscous forces. Re is defined as

$$\mathrm{Re} = \frac{\rho u L}{\mu} \quad \text{(S1)}$$

where ρ is the density of the fluid (kg/m^3), u is the flow velocity (m/s), L is the characteristic linear dimension (m), and μ is the dynamic viscosity of the fluid (Pa·s). For the experiments presented here, we can approximate the fluid as pure deionized water, i.e. $\rho = 997$ kg/m^3 and $\mu = 10^{-3}$ Pa·s. Applying the threshold velocity measured in the frame-by-frame cell tracking ($u = 34.2$ μm/s) and $L = 220$ μm as the diameter of the plasmonic optical fiber, **Eq. (S1)**) becomes

$$\mathrm{Re} = \frac{(997\mathrm{kg/m^3})(34.2\times10^{-6}\mathrm{m/s})(220\times10^{-6}\ \mathrm{m})}{10^{-3}\ \mathrm{Pa\cdot s}} = 7.501 \times 10^{-3} \quad \text{(S2)}$$

which gives Re smaller than unity, verifying that the presented system is within the laminar flow regime (Re < 2300) [1]. When Re ≪ 1, the motion of particles in a suspension (i.e. *E. coli* suspension in this work) nearly perfectly follows Stokes' law.

2. Solution of motion equations for calculation of the thermophoretic trapping force

For the case of thermophoretic trapping of a single bacterium (i.e. the situation depicted in **Fig. 4**(e)), the motion equations in each direction are defined as shown below (where *t*, *d*, *l*, *v* and a represent time, distance, length, velocity and acceleration respectively).

y-direction: l = 176.75 μm (max. traveling distance = diagonal length of 125 × 125 μm^2)

$$t = \frac{l}{v_y} = \frac{176.75\ \mu\text{m}}{34.2\ \mu\text{m/s}} = 5.18\ \text{sec} \tag{S3}$$

x-direction: d_{bl} = 25.7 μm; t = 5.178 sec; v_{x0} = 0 μm/s

$$d_{\text{bl}} = \frac{1}{2}|a_x|t^2, \ \ \therefore |a_x| = 1.91\ \mu\text{m/s}^2 \tag{S4}$$

$$v_x^2 - v_{x0}^2 = 2|a_x|d_{\text{bl}}, \ \therefore v_x = 9.91\ \mu\text{m/s} \tag{S5}$$

Therefore, the Stokes drag force is

$$F_{\text{D}} = 3\pi\mu_{\text{w}}d_{\text{p}}v_x = 100\ \text{fN} \tag{S6}$$

where $\mu_{\text{w}} = 10^{-3}$ Pa·s and $d_{\text{p}} = d_{E.coli} = 1.075 \times 10^{-6}$ m.

As discussed in the main text, to securely trap a bacterium, we require that the thermophoretic trapping force (F_{Th}) exceeds the drag force. Thus,

$$|F_{\text{Th}}| > 100\ \text{fN} \tag{S7}$$

3. Descriptions of supplementary videos

Visualization 1. E. coli accumulation on planar substrates

This video demonstrates the plasmo-thermal *E. coli* accumulation effect on planar substrates under a Raman imaging microscope (DXR2xi, Thermo Fisher Scientific, USA). Data was acquired using a 785 nm excitation laser, a 50 µm slit aperture, a 50× long working distance objective (Olympus, Japan), 5 mW of excitation power, a 2.5 ms (400 Hz) excitation time, and 10 averaging scans. The microscope's white light illumination mode was used to record the video clips. These were taken using a screen recording software (Bandicam, Bandicam Company, USA) before and after 4 minutes of laser exposure at a spot on a plain gold surface and on several hexagonal single voxel arrays (HSVAs) with spacing distances (a) of 300/700/800/1,100/1,400 nm. Due to the interlock mechanism of the imaging Raman microscope, live imaging was not available during laser exposure. The video starts from the plain gold surface and then shows the HSVAs in order of increasing spacing distance. The video clips of all the cases after the exposure are collated at the end of the video to allow intuitive comparison. The video demonstrates that the highest number of swimming *E. coli* were trapped/accumulated on the HSVA with a = 700 nm.

Visualization 2. E. coli accumulation and diffusion at the plasmonic optical fiber tip

This video demonstrates *E. coli* accumulation and diffusion at the plasmonic optical fiber tip as the laser (785 nm) was switched on and off. The video was recorded using a 10× objective lens and a camera sensor that acted as a 10× eyepiece lens (yielding 100× total magnification, see **3. Methods**).

Visualization 3. E. coli accumulation and diffusion at the plasmonic optical fiber tip—low magnification

This video demonstrates *E. coli* accumulation and diffusion at the plasmonic optical fiber tip as the laser was switched on and off. The video was recorded using a 4× objective lens (yielding 40× total magnification).

Visualization 4. Switching of convection current flow direction upon shifting of focal plane

This video shows the change in the direction of the convection current flow as the focal plane of the microscope was moved up and down. The change in the flow direction as the focal plane was shifted confirmed that a toroidal convection current had been generated.

4. Supplementary figures

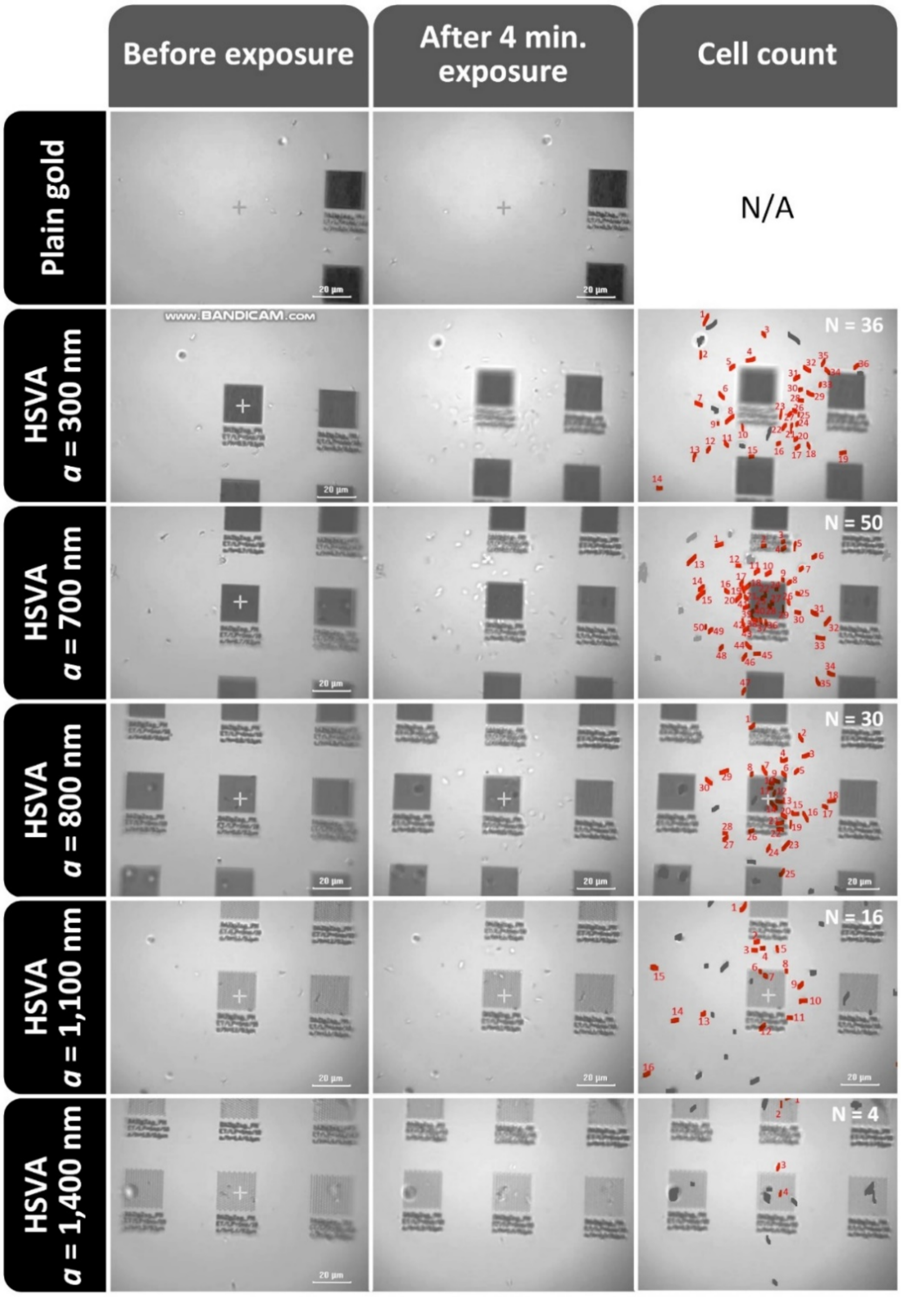

Fig. S1. Plasmo-thermal accumulation of swimming *E. coli* on planar substrates (snapshots from Visualization 1). Snapshots of the video clips before (left column) and after (middle column) 4 minutes of laser exposure on different planar substrates (a plain gold surface and hexagonal single voxel arrays (HSVAs) with spacing distances of *a* = 300/700/800/1,100/1,400 nm). The accumulated *E. coli* cells were manually counted (right column). Red—cells added after exposure; gray—cells observed since before exposure (*N*: total number of accumulated *E. coli* cells).

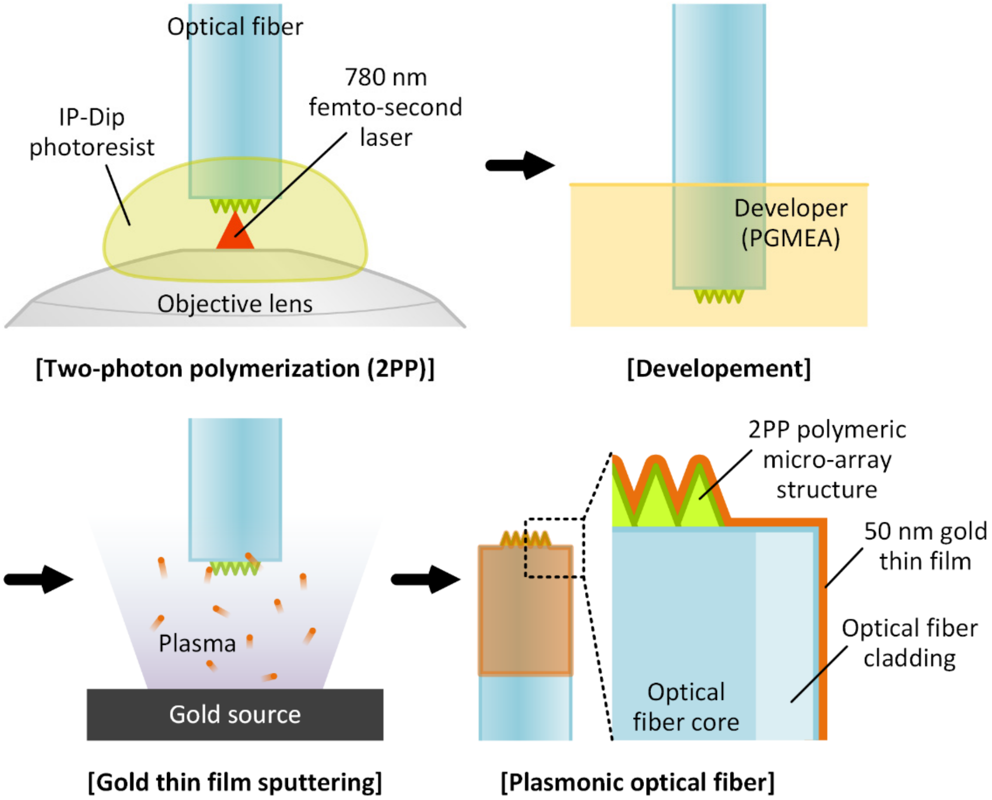

Fig. S2. Fabrication process of the plasmonic optical fiber using two-photon polymerization (2PP) and gold sputtering.

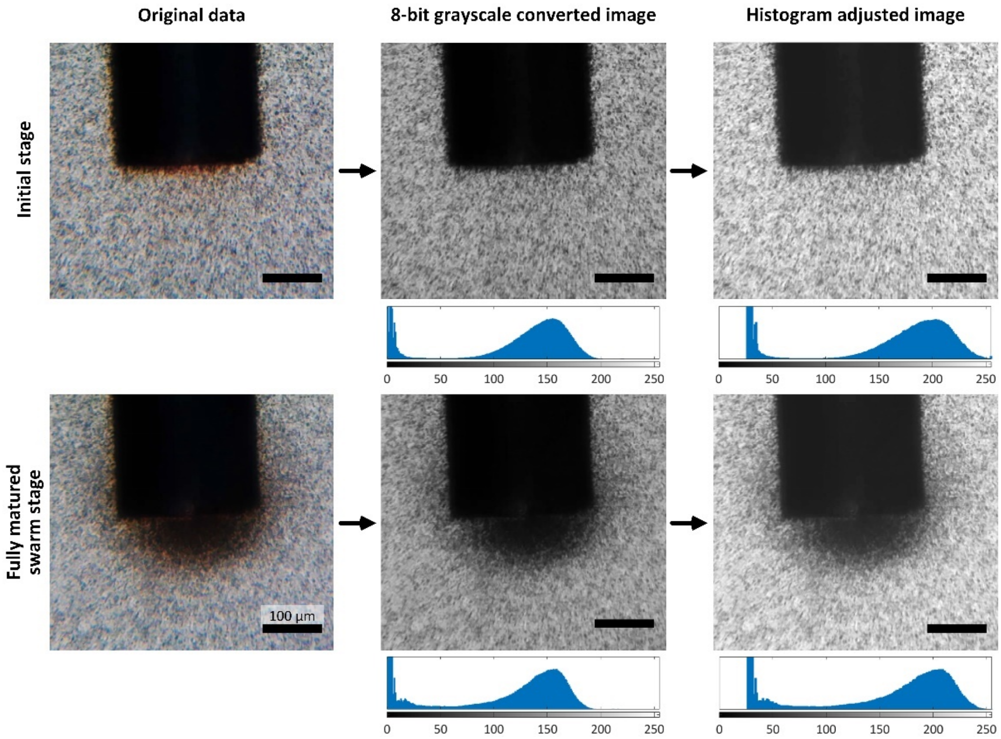

Fig. S3. Step-by-step demonstration of the image processing protocol for the initial stage of the accumulation and the fully matured swarm stage. The image processing involved conversion of 24-bit color format to 8-bit grayscale followed by brightness/contrast adjustment by re-scaling the pixel intensity histogram ranges from 0-198.9 to 25.5-255 (blue histogram plots underneath the corresponding images).

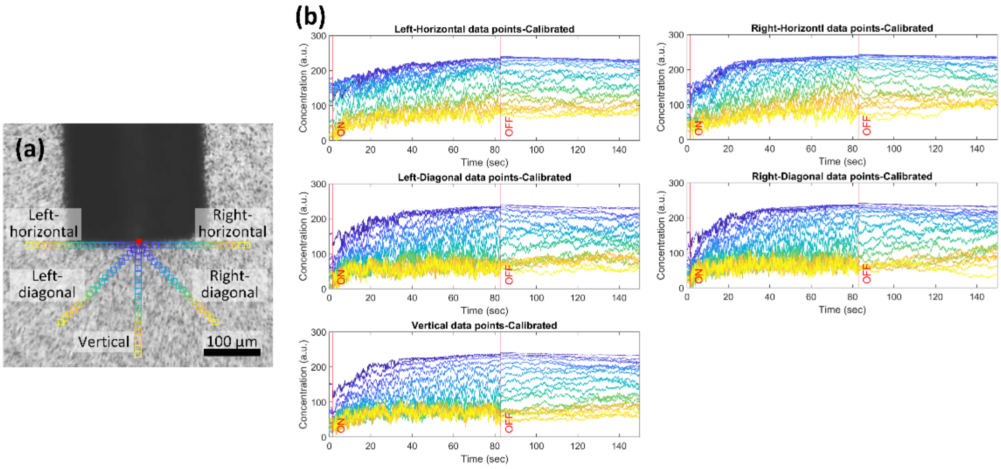

Fig. S4. (a) Optical microscope image of plasmonic optical fiber probe in *E. coli* solution (the frame before the laser was switched on). 20 square regions of interest (ROIs; colored squares; individual ROI area = 10×10 µm^2) were generated in five different directions from the center of the tip (red dot). Scale bar: 100 µm. (b) Concentration change over time at each ROI in the five different directions illustrated in (a).

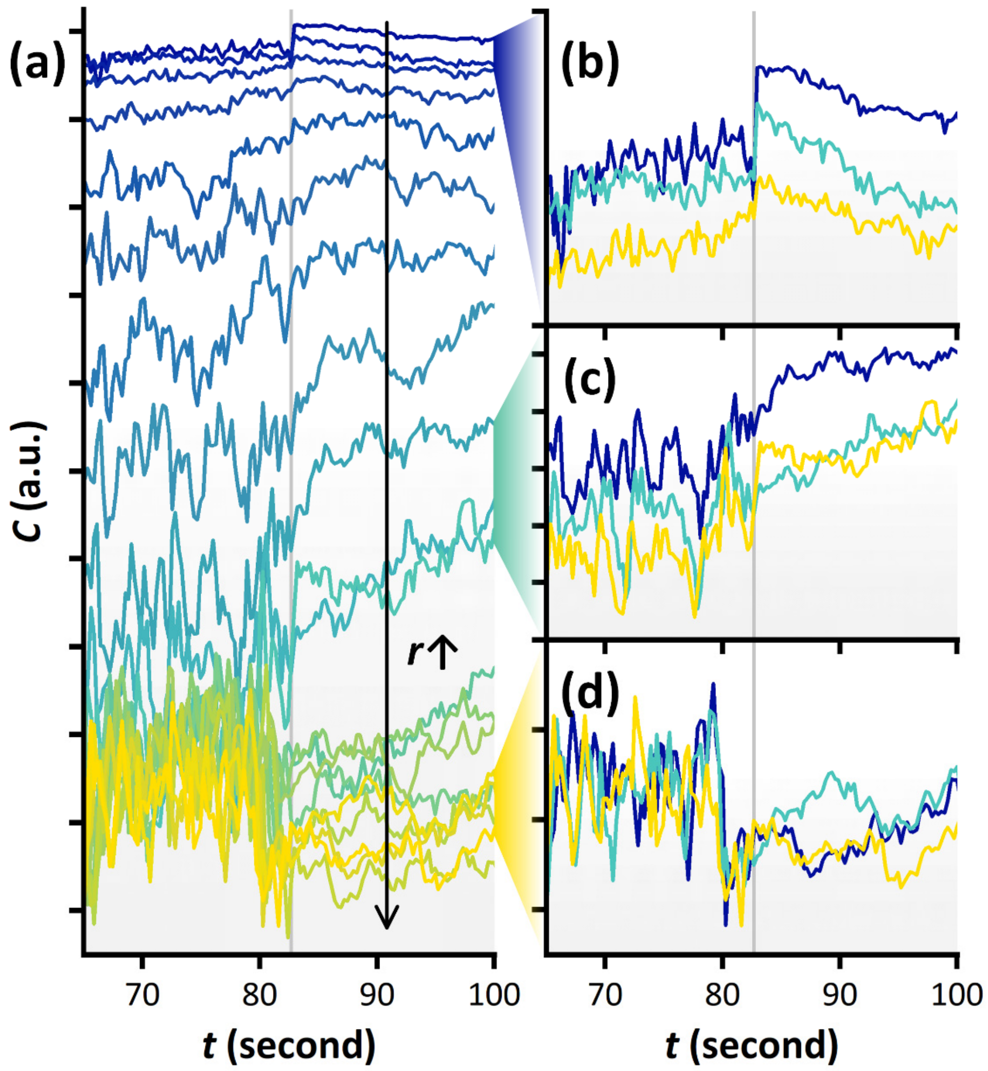

Fig. S5. Zoomed in raw *C*-*t* plots from the ROIs in the vertical direction indicated in Fig. S4(a) near the instant at which the laser was switched off (t = 82.7 sec; gray line). (a) Plots from all of the 20 ROIs, (b) from three near-range ROIs at r = 5/15/25 μm, (c) from three mid-range ROIs at r = 85/95/105 μm, and (d) from three far-range ROIs at r = 175/185/195 μm. A step response was observed at near ranges (b) at the point at which the laser was switched off indicating an instantaneous increase in concentration. This effect was in fact due to the removal of laser illumination resulting in a sudden brightness drop (which generated an anomalous increase in concentration due to calculation of the concentration using the equation $C = 255 - I$). This effect was not observed at greater distances (c, d) where the brightness drop following removal of laser illumination was less pronounced. A noise reduction was observed after the laser was switched off at all distance ranges. This was attributed to the more tranquil manner of bacterial motion in the diffusion phase without the vigorous plasmo-thermal convection current.

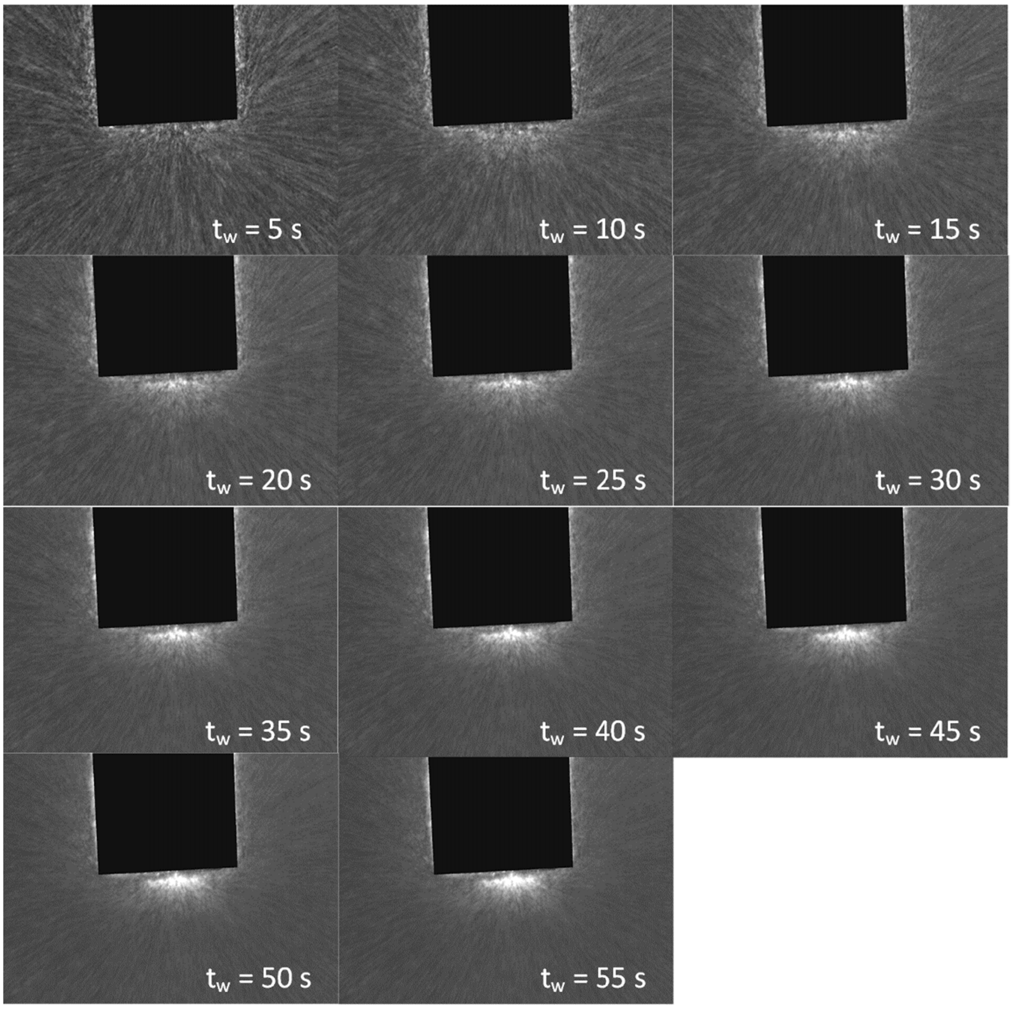

Fig. S6. Temporal speckle contrast (K) images with different time window (t_w) values, which were used to find the optimal t_w value. Spatial resolution was lost as t_w increased. Thus, for the analysis presented in the main text, a t_w value of 15 s was used in order to minimize noise while maintaining an acceptable spatial resolution.

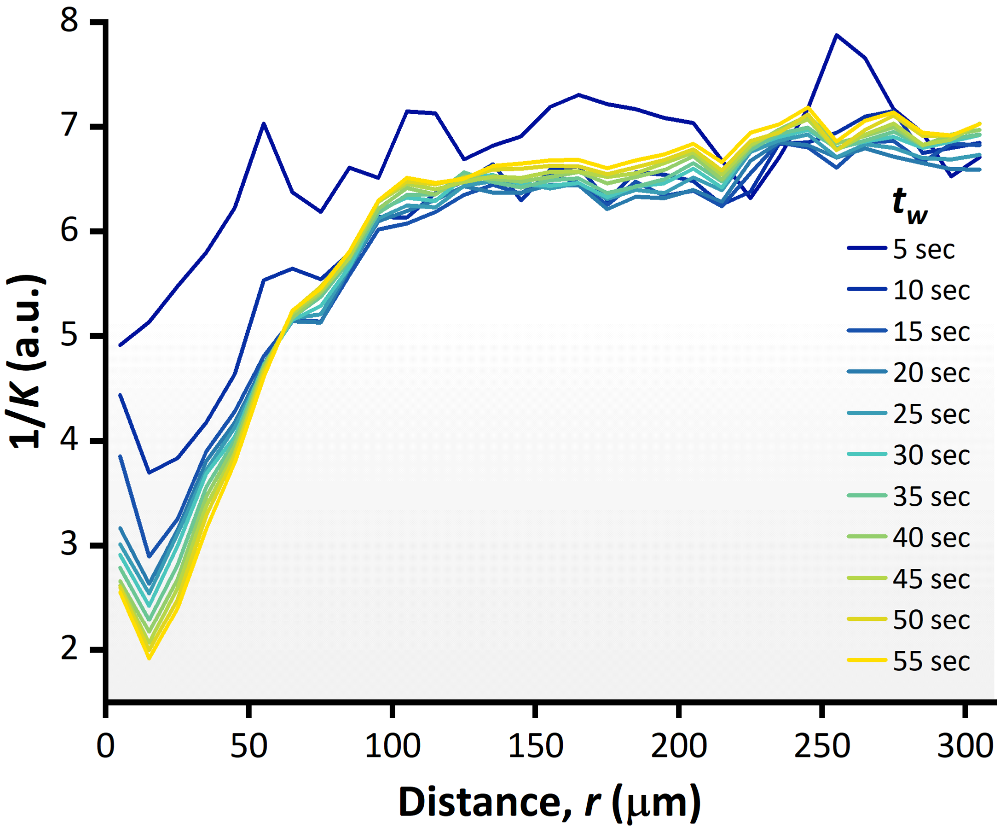

Fig. S7. Inverse *K* vs. distance (*r*) plots for varying t_w, averaged across five different directions from the center of the plasmonic optical fiber tip (see Fig. 5(d) inset). The noise level decreased as t_w increased.

References

1. H. Schlichting and K. Gersten, *Boundary-Layer Theory*, 9th ed. (Springer Berlin Heidelberg, 2016).
